# Quantitative trait loci and genomic prediction for grain sugar and mineral concentrations of cowpea [*Vigna unguiculata* (L.) Walp.]

**DOI:** 10.1038/s41598-024-55214-2

**Published:** 2024-02-25

**Authors:** Bao-Lam Huynh, James C. R. Stangoulis, Tri D. Vuong, Haiying Shi, Henry T. Nguyen, Tra Duong, Ousmane Boukar, Francis Kusi, Benoit J. Batieno, Ndiaga Cisse, Mouhamadou Moussa Diangar, Frederick Justice Awuku, Patrick Attamah, José Crossa, Paulino Pérez-Rodríguez, Jeffrey D. Ehlers, Philip A. Roberts

**Affiliations:** 1 Department of Nematology, University of California, Riverside, CA USA; 2 College of Science and Engineering, Flinders University, Bedford Park, SA Australia; 3 Division of Plant Science and Technology and National Center for Soybean Biotechnology, University of Missouri, Columbia, MO USA; 4 International Institute of Tropical Agriculture, Kano, Nigeria; 5 CSIR-Savanna Agricultural Research Institute, Tamale, Ghana; 6 Institut de l’Environnement et de Recherches Agricoles, Kamboinse, Burkina Faso; 7 Institut Senegalais de Recherches Agricoles, Thies, Senegal; 8 International Maize and Wheat Improvement Center, Mexico City, Mexico; 9 Colegio de Postgraduados, Montecillo, Edo. de Mexico Mexico; 10 Bill and Melinda Gates Foundation, Seattle, WA USA

**Keywords:** Mass spectrometry, Genetic mapping, Agricultural genetics, Genetic markers, Quantitative trait

## Abstract

Development of high yielding cowpea varieties coupled with good taste and rich in essential minerals can promote consumption and thus nutrition and profitability. The sweet taste of cowpea grain is determined by its sugar content, which comprises mainly sucrose and galacto-oligosaccharides (GOS) including raffinose and stachyose. However, GOS are indigestible and their fermentation in the colon can produce excess intestinal gas, causing undesirable bloating and flatulence. In this study, we aimed to examine variation in grain sugar and mineral concentrations, then map quantitative trait loci (QTLs) and estimate genomic-prediction (GP) accuracies for possible application in breeding. Grain samples were collected from a multi-parent advanced generation intercross (MAGIC) population grown in California during 2016–2017. Grain sugars were assayed using high-performance liquid chromatography. Grain minerals were determined by inductively coupled plasma–optical emission spectrometry and combustion. Considerable variation was observed for sucrose (0.6–6.9%) and stachyose (2.3–8.4%). Major QTLs for sucrose (*QSuc.vu-1.1*), stachyose (*QSta.vu-7.1*), copper (*QCu.vu-1.1*) and manganese (*QMn.vu-5.1*) were identified. Allelic effects of major sugar QTLs were validated using the MAGIC grain samples grown in West Africa in 2017. GP accuracies for minerals were moderate (0.4–0.58). These findings help guide future breeding efforts to develop mineral-rich cowpea varieties with desirable sugar content.

## Introduction

Soluble carbohydrates including mono-, di-, and short-chain oligosaccharides are present in the edible parts of many crops and elicit a sweet taste when consumed^1^. In cowpea (*Vigna unguiculata* L. Walp.) and other food legumes, short-chain oligosaccharides include galacto-oligosaccharides (GOS) or raffinose family oligosaccharides, such as raffinose and stachyose^2–4^. Since humans do not have enzymes to break down GOS, they bypass digestion in the small intestine and become fermented by colonic bifidobacteria and lactobacilli in the large intestine to produce short-chain fatty acids that provide numerous health benefits^5^. This selective stimulation of microbial growth also helps reduce colonization of pathogenic bacteria and viruses in the gut^6^. The prebiotic fermentation also reduces pH in the gut environment and thus increases the guts ability to absorb more nutrients from diets, particularly calcium and iron^7^. However, the fermentation of GOS can also cause intestinal discomfort when excess intestinal gas is produced, which leads to bloating and flatulence^8^; the latter is considered embarrassing in many cultures, thus reducing legume consumption by people with sensitive gastrointestinal systems. Thus, developing new improved varieties of food legumes with a sweet taste and moderate GOS content can promote their consumption. Increased cowpea consumption would lead to better nutrition in populations and greater demand for the commodity. This is especially true for cowpea, a vitally important crop for food security in West Africa where it provides a main source of protein and essential minerals that complement cereals in the human diet^9–11^.

Developing “sweet” cowpea varieties has long been a breeding objective, following the sweet-trait discovery in a Cameroonian cowpea breeding line in the late 1990s^12,13^. Likewise, breeding for nutrient-dense cowpea varieties has also been a focus because of the considerable genetic variation observed in global cowpea germplasm collections^9^; the maximum concentrations of grain iron, zinc and some other minerals were more than double those of accessions with the lowest amounts. Breeding can also make use of greater understanding of trait inheritance. However, information on the inheritance of the sweet trait and grain mineral accumulation and their relationship with agronomic traits in cowpea is still lacking. Previous studies revealed multiple quantitative trait loci (QTLs) affecting grain sucrose, raffinose and stachyose contents in soybean^14^ and grain iron and zinc accumulation in common bean^15^. Such genetic control may also exist in related grain legumes from the Fabaceae family including cowpea. Understanding of genetic factors and relationships between grain sugar and mineral accumulation in cowpea would enable improving them together through marker-assisted breeding strategies.

In this study, we aimed to detect and map QTLs for grain soluble sugars and mineral accumulation using a multi-parent advanced generation intercross (MAGIC) cowpea population of 305 recombinant inbred lines (RILs) derived from eight founder parents, including a sweet cowpea landrace from Africa^16,17^; they are genetically diverse and thus expected to contribute different favorable alleles to each nutritional trait. We also aimed to estimate the accuracies of genomic prediction (GP) for these traits using the cowpea MAGIC as a training population. Using GP in selection does not require QTL information^18^, so it can be suitable for improving traits that lack QTLs or are controlled mostly by minor effect QTLs. The cowpea MAGIC population was comprehensively genotyped and comprised individuals which were carefully selected based on genome-wide marker diversity, so interference in GP analysis by kinship and population structure would be minimal. Correlations between nutritional traits, flowering time and seed size were also examined for application in multi-trait selection. The findings will help guide genetic biofortification efforts to develop elite cowpea varieties with added nutritional values, desirable taste and greater digestibility.

## Results

### Phenotypic variation

**Figure 1:**
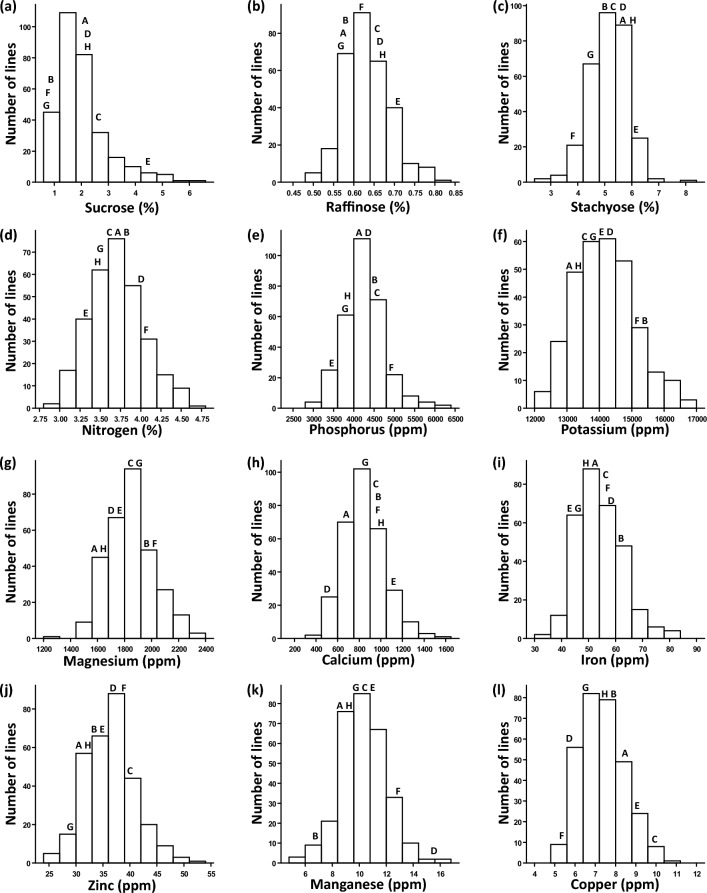
Frequency distribution for mean concentration of (**a–c**) grain sugars and (**d–l**) mineral nutrients measured in the cowpea MAGIC population grown in Thermal, California in 2016 and 2017. Values for the eight founder parents are indicated by capital letters: A, IT89KD-288; B, IT84S-2049; C, CB27; D, IT82E-18; E, Suvita-2; F, IT00K-1263; G, IT84S-2246; H, IT93K-503-1. Significant differences (*P* < 0.001) exist among the MAGIC and parental lines.

The concentration of grain sugars and minerals varied considerably in the MAGIC RILs and parents grown at the University of California Riverside Coachella Valley Agricultural Research Station (CVARS) during 2016–2017. Stachyose showed the highest concentration, on average 5.1% (percentage of grain dry weight), ranging from 2.3 to 8.4%, followed by sucrose (0.6–6.9%, average 2%) and raffinose (0.5–0.9%, average 0.6%). Among grain macronutrients, nitrogen was most concentrated (2.5–4.8%, average 3.7%), followed by potassium (1.14–1.77%, average 1.42%), phosphorus (0.27–075%, average 0.43%), magnesium (0.12–0.24%, average 0.18%), and calcium (0.04–0.18%, average 0.09%). Among micronutrients, iron showed the highest concentration, on average 54.3 ppm (parts per million), ranging from 32.5 to 91 ppm, followed by zinc (32.2–71.1 ppm, average 36.5 ppm), manganese (5.2–20.2 ppm, average 10.2 ppm) and copper (4.7–12.8 ppm, average 7.3 ppm). Nitrogen, magnesium and manganese showed a normal distribution based on the Shapiro–Wilk test, while other nutrients were skewed toward lower or higher values. Transgressive segregation, in which phenotypic values of many progeny were outside the range of parental phenotypes, was observed for most grain nutrients, except sucrose, manganese and copper (Fig. 1).

**Figure 2:**
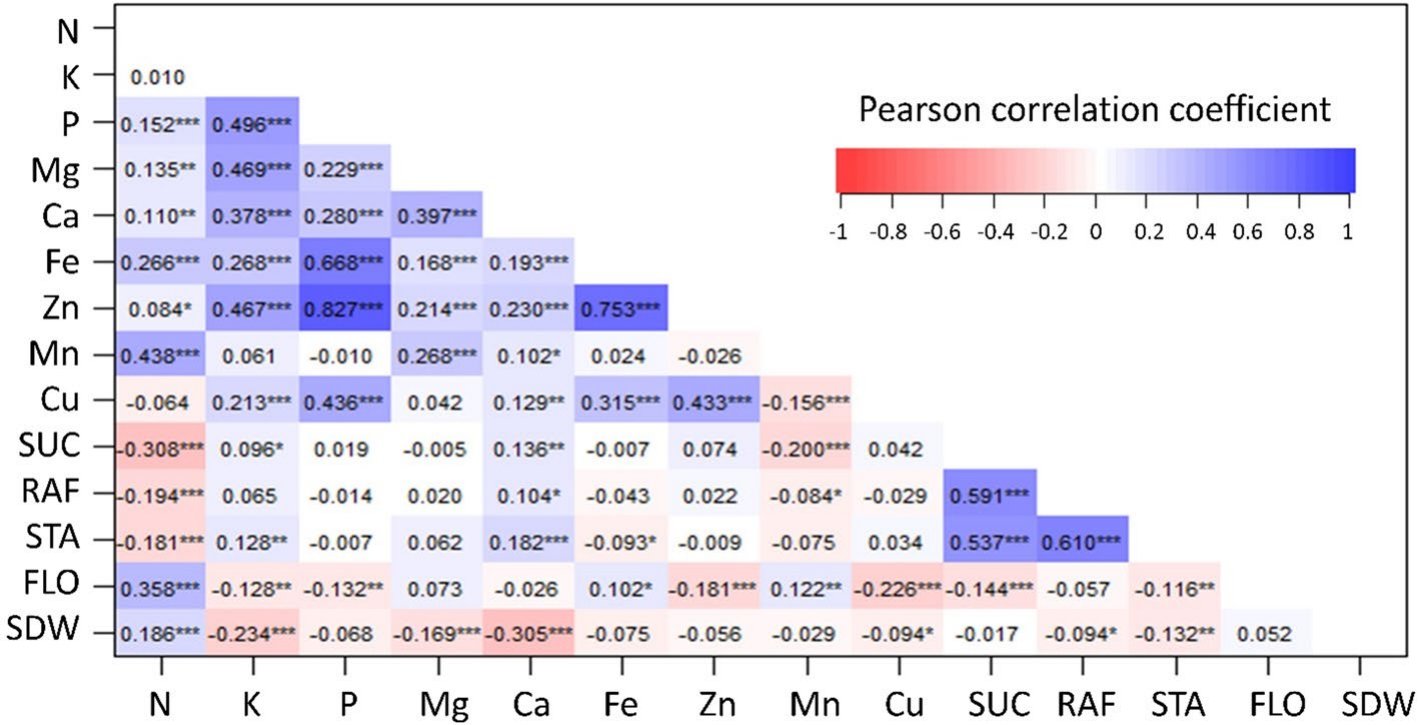
Phenotypic correlations among grain concentrations of minerals and sugars (SUC: sucrose, RAF: raffinose, STA: stachyose) and agronomic traits (FLO: flowering time, SDW: seed size) in the cowpea MAGIC population grown in Thermal, California in 2016 and 2017. The asterisks (*, **, ***) indicate significance at the 0.05, 0.01, and 0.001 levels, respectively.

Significant (*P* < 0.001) positive correlations appeared between the 2016 and 2017 data of the same nutrients, among which sucrose showed the strongest correlation (*r* = 0.86) (Supplemental Table S1). There were also significant correlations among nutrient concentrations (Fig. 2). Most notably, iron and zinc were strongly and positively correlated with each other (*r* = 0.75, *P* < 0.001) and with phosphorus (*r* = 0.67 and 0.83, *P* < 0.001). The three soluble sugars (sucrose, raffinose and stachyose) were also positively correlated with each other (*r* > 0.54, *P* < 0.001) but negatively correlated with nitrogen concentration (*r* < -0.18, *P* < 0.001). Flowering time and seed size had significant (*P* < 0.001) positive correlations with nitrogen concentration (*r* = 0.36 and 0.19, respectively) but negative or neutral correlations with other mineral concentrations.

### QTL detection

**Figure 3:**
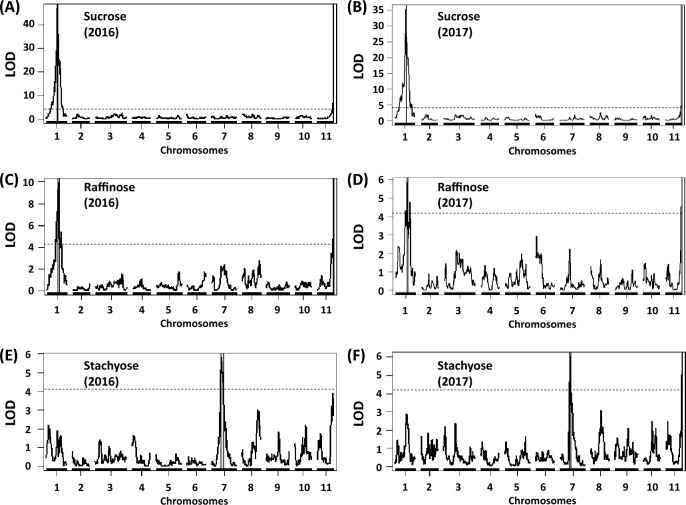
Chromosomal regions associated with grain sucrose, raffinose and stachyose concentrations measured in the cowpea MAGIC population grown in Thermal, California in 2016 (left) and 2017 (right). Vertical lines flanking QTL peaks indicate 1-LOD support intervals. Dashed lines indicate the significance threshold at 7.56 × 10^–5^ (LOD = 4.12) using empirical null simulations (n = 1000, *P* = 0.05).

Two QTLs affecting sucrose concentrations were consistently identified using data from the two phenotyping trials at CVARS in 2016 and 2017 (Fig. 3A,B, Table S2). The major QTL, *QSuc.vu-1.1*, was located on chromosome 1 of the cowpea MAGIC genetic map, explaining approximately 38–48% of the total phenotypic variation. The high-sucrose allele was contributed from Suvita-2. The minor QTL with a lower additive effect, *QSuc.vu-11.1*, was located on chromosome 11, explaining 9–13% of the total phenotypic variation, with the high-sucrose alleles contributed from Suvita-2 and IT89KD-288. These two QTLs also coincided with *QRaf.vu-1.1* and *QRaf.vu-11.1* affecting raffinose concentrations, explaining 8–15% of the total phenotypic variation (Fig. 3C,D, Table S2). The minor QTL for sucrose was also co-located with the QTL *QSta.vu-11.1* affecting stachyose concentrations and explaining approximately 9% of the total phenotypic variation in 2017. Another QTL located on chromosome 7, *QSta.vu-7.1*, was also associated with the stachyose concentration, explaining approximately 11% of the total phenotypic variation in both 2016 and 2017 seasons, with the high-stachyose alleles contributed from IT89KD-288, IT84S-2049, IT82E-18, IT00K-1263 and IT93K-503-1 (Fig. 3E,F, Table S2).

**Figure 4:**
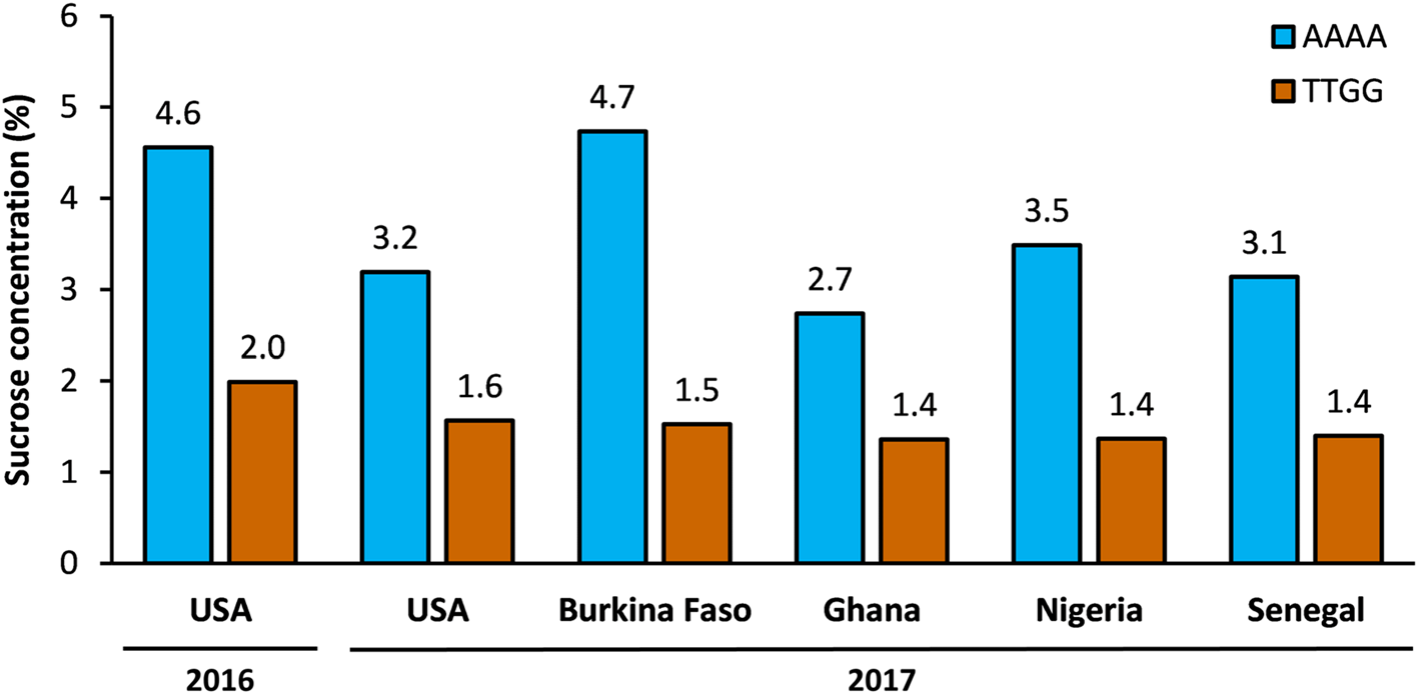
Mean grain sucrose concentrations of two genotypic classes of MAGIC lines with contrasting SNP haplotypes defined by two markers 2_32637 and 2_40402 flanking *QSuc.vu-1.1*: AAAA (lines homozygous for the high-sucrose haplotype from Suvita-2, blue bars) and TTGG (lines homozygous for the low-sucrose haplotype of the other parents, orange bars). Values shown for USA are mean values from individual lines. Values shown for Burkina Faso, Ghana, Nigeria and Senegal are based on the assessment of grain samples bulked within genotypic classes. Significant differences (*P* < 0.05) exist between genotypic classes.

**Figure 5:**
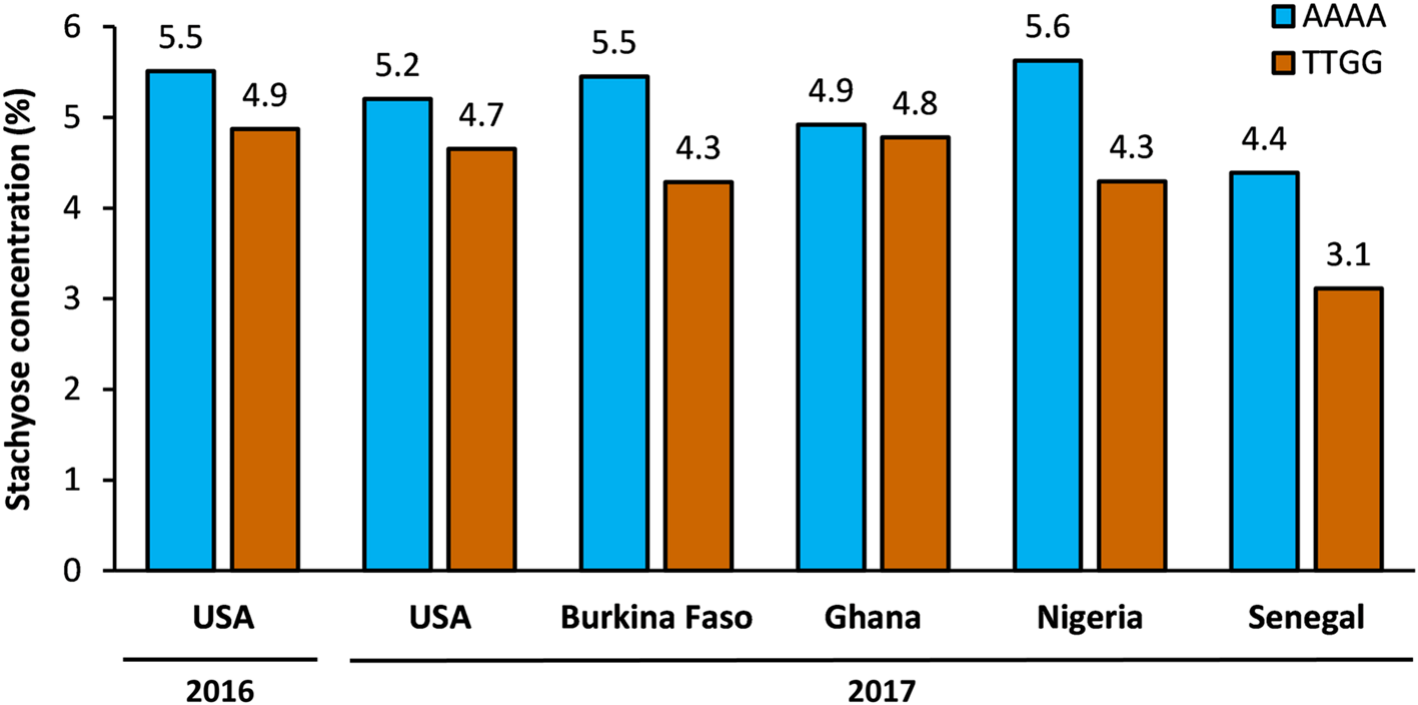
Mean grain stachyose concentrations of two genotypic classes of MAGIC lines with contrasting SNP haplotypes defined by two markers 1_0585 and 2_01076 flanking *QSta.vu-7.1*: AAAA (lines homozygous for the high-stachyose haplotype from IT89KD-288, IT84S-2049, IT82E-18, IT00K-1263 and IT93K-503-1, blue bars) and TTGG (lines homozygous for the low-stachyose haplotype from Suvita-2 and IT84S-2246, orange bars). Values shown for USA are mean values from individual lines. Values shown for Burkina Faso, Ghana, Nigeria and Senegal are based on the assessment of grain samples bulked within genotypic classes. Significant differences (*P* < 0.05) exist between genotypic classes.

The allelic effects of *QSuc.vu-1.1* and *QSta.vu-7.1* were validated using grain samples from four field trials across West Africa (Burkina Faso, Nigeria, Senegal and Ghana). At *QSuc.vu-1.1*, two markers (2_32637 and 2_40402) distinguishing between the high-sucrose allele donor (Suvita-2) and the other parents were used to form two genotypic classes. Sucrose concentrations measured for grain bulks of MAGIC lines carrying the Suvita-2 haplotype were significantly (*P* < 0.05) higher than those carrying other parental haplotypes (Fig. 4). Likewise, at *QSta.vu-7.1*, two markers (1_0585 and 2_01076) distinguishing between the high-stachyose allele donors (IT89KD-288, IT84S-2049, IT82E-18, IT00K-1263 and IT93K-503-1) and the other parents were used to establish two genotypic classes. Stachyose concentrations measured for grain bulks of MAGIC lines carrying the high-stachyose haplotype were significantly (*P* < 0.05) higher than those carrying the low-stachyose haplotype (Fig. 5). These significant effects were based on analysis of variance in which QTL haplotypes and field locations across West Africa were considered as fixed and random factors, respectively. On average, the favorable haplotypes at *QSuc.vu-1.1* and *QSta.vu-7.1* contributed to an increase of 2% and 1% (of grain dry weight) respectively in grain sucrose and stachyose concentrations compared to the unfavorable haplotypes.

**Figure 6:**
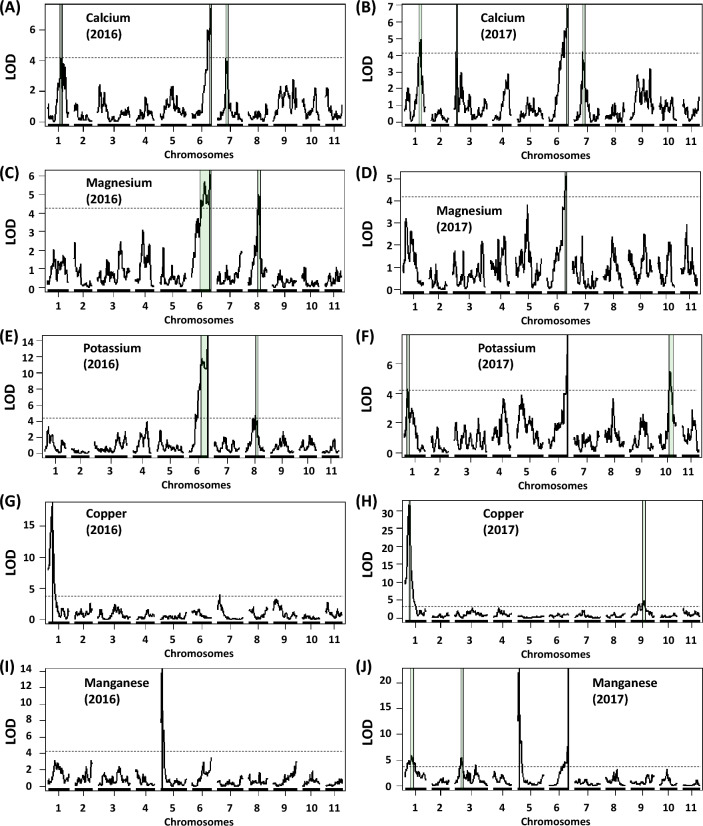
Chromosomal regions associated with grain calcium, magnesium, potassium, copper and manganese concentrations measured in the cowpea MAGIC population grown in Thermal, California in 2016 (left) and 2017 (right). Vertical lines flanking QTL peaks indicate 1-LOD support intervals. Dashed lines indicate the significance threshold at 7.56 × 10^–5^ (LOD = 4.12) using empirical null simulations (n = 1000, *P* = 0.05).

Several QTLs affecting grain mineral concentrations were also detected and mapped to multiple chromosomal regions, none of which were co-located with the sugar QTLs (Fig. 6, Table S2). Three QTLs affecting Ca concentrations were consistently identified using both 2016 and 2017 CVARS phenotypic data sets (Fig. 6A,B), located on chromosomes 1, 6 and 7, each explaining 8–14% of the total phenotypic variation. Of these, the QTL on chromosome 6 (*QCa.vu-6.1*) also coincided with those affecting Mg and K concentrations (Fig. 6C–F). Most notably, QTLs with major effects were identified for Cu on chromosome 1 (*QCu.vu-1.1*, Fig. 5G,H) and Mn on chromosome 5 (*QMn.vu-5.1*, Fig. 6I,J), explaining up to 36% and 28% of the total phenotypic variation, respectively. Other QTLs for Fe, Zn and N concentrations showed minor effects, and they were detected only in the 2016 or 2017 environment (Table S2).

QTLs for flowering time and seed size were mapped in similar genome regions reported by Huynh et al.^16^. Five QTLs affecting flowering time in 2016 were mapped on chromosomes 1, 4, 5, 9 and 11; of these, QTLs on chromosomes 4 and 9 also affected flowering time in 2017 (Table S2). All QTLs showed minor effects, each explaining less than 18% of total phenotypic variance. The parent CB27 consistently contributed to the early-flowering allele at each QTL. The QTLs on chromosomes 1, 5 and 9 were co-located with those affecting Ca, Mn and Fe concentrations, respectively. For seed size, one minor and one major QTL were identified on chromosomes 6 and 8, respectively (Table S2). The major QTL explained up to 38% of total phenotypic variance, with favorable (large seed) alleles contributed from IT82E-18 and IT00K-1263. This QTL was co-located with those affecting N and P concentrations. The other QTL with minor effect was located on chromosome 6, explaining approximately 9% of total phenotypic variance, with the favorable allele contributed from IT89KD-288 (Table S2). This QTL was also co-located with those affecting Ca, Mg, Mn, N and K concentrations.

The wide ranges and transgressive variation of grain sugar and mineral concentrations support the presence of multiple QTLs detected, with favorable alleles contributed from different founder parents. Traits affected by major QTLs, such as glucose and stachyose concentrations, seemed to skew toward lower or higher phenotypic values while those affected by minor QTLs exhibited a normal phenotypic distribution (Fig. 1). Common QTLs found among traits and years were also consistent with their positive correlation (Fig. 2 and Supplemental Table S1). Of these, one common QTL region for sucrose, raffinose and stachyose was identified on chromosome 11, in addition to a specific QTL for stachyose on chromosome 7, and the other for sucrose and raffinose on chromosome 1 (Fig. 3). This genetic relationship reflects the FOS biosynthetic pathways to be mentioned hereafter in the discussion.

### Genomic prediction

**Figure 7:**
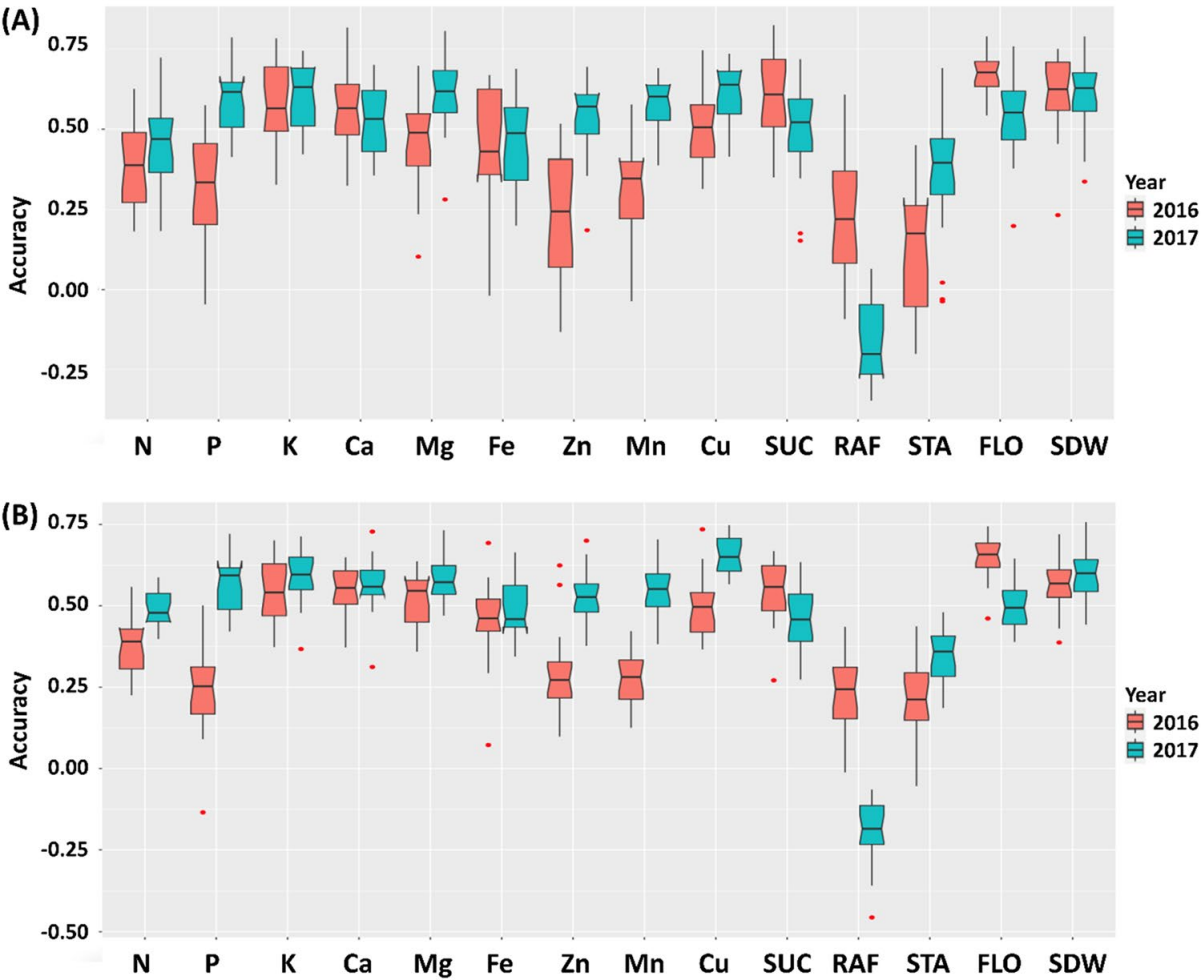
Genomic prediction accuracies using Bayesian Ridge Regression model for concentrations of grain minerals, soluble sugars (SUC: sucrose, RAF: raffinose, STA: stachyose), and agronomic traits (FLO: flowering time, SDW: seed size) measured in the cowpea MAGIC population grown in Thermal, California in 2016 (orange) and 2017 (cyan). Two cross-validation settings are presented: (**A**) Testing/Training = 1/9, and (**B**) Testing/Training = 1/4.

The GP accuracy (i.e., correlations between predicted and actual phenotypic values) differed significantly (*P* < 0.001) among nutrient concentrations measured in the MAGIC population planted at CVARS in 2016 and 2017 based on two cross validation schemes using Bayesian Ridge Regression (Fig. 7). In the 1/9 scheme (Fig. 7A), 90% of lines were used to predict the remaining 10%. In the 1/4 scheme (Fig. 7B), 80% of lines were used to predict the remaining 20%. There was no difference (*P* > 0.05) between these two schemes, although data derived from the 1/9 setting appeared more dispersed. On average, seed size and flowering time had the highest GP accuracy (0.59), followed by K (0.58), Cu (0.57), Ca (0.55), Mg (0.54), sucrose (0.53), Fe (0.46), N (0.43), P (0.43), Mn (0.43), Zn (0.40), stachyose (0.26) and raffinose (0.02). Overall, the trial in 2017 with an augmented row-column design gave a significantly (*P* < 0.001) better prediction (0.48 on average) than the single-plot design trial in 2016 (0.43 on average).

## Discussion

The moderate GP accuracies (0.40–0.58) for cowpea mineral concentrations (Fig. 7) overlap those reported recently for wheat^19^ and rice^20^. This indicates the potential of GP in genetic biofortification to enrich cowpea grain with nutritional elements. Taking all traits together there appears to be a correlation between QTL information and GP accuracies. Traits with major effect QTLs (e.g. seed size, sucrose and copper) and those with many minor effect QTLs (e.g., calcium, nitrogen and iron) had better GP accuracies than traits with fewer minor-effect QTLs (Fig. 7 and Table S2). This supports the advantage of GP in which breeding values are estimated using all genome-wide markers^21^ that would also capture QTL effects through linkage disequilibrium.

The presence of common and specific QTLs for different grain sugars (Fig. 3) reflects the FOS biosynthetic pathways^22^ in which sucrose serves as a starting material, where enzymes subsequently add galactose units to sucrose, yielding raffinose and other longer FOS polymers. Sequencing of MAGIC RILs and parents carrying low- vs high-concentration alleles may help reveal gene polymorphisms underlying differential sugar accumulation in the cowpea grain. Sequence assembly and QTL candidate-gene identification would be supported by the cowpea reference genome^23^ and the homolog genes of common bean available on Phytozome^24^.

It seems likely that sweetness and mineral accumulation are under different genetic controls, because none of the sugar QTLs coincided with those affecting mineral accumulation. The two major QTLs for grain sucrose (*QSuc.vu-1.1*) and stachyose (*QSta.vu-7.1*) appeared stable across environments based on results from the validation of QTL allelic effects (Figs. 4, 5). Previous studies also revealed QTLs with major effects on sugar accumulation in the grain of other crops such as wheat^25^ and soybean^14^. In contrast, the inheritance of grain mineral accumulation, except Cu and Mn, appears more complex with many QTLs with small effects (Fig. 6 and Table S2). Previous studies also reported QTLs with small effects on mineral accumulation in the grain of other crops such as rice^26^ and common bean^15^. The challenge in detecting QTLs with major effects on grain mineral accumulation could be attributed to soil spatial variation, whereas sugar biosynthesis might be influenced mainly by sunlight through photosynthesis in addition to carbon dioxide, temperature and water availability as seen in common bean^27,28^; these factors would be more uniformly distributed than soil in the field under sub-surface irrigation at CVARS. Indeed, more QTL peaks and better GP accuracies were discovered for grain minerals from the trial at CVARS in 2017, where data were corrected taking into account spatial variation, compared to the non-replicated trial in 2016 (Figs. 6, 7, Table S2).

The information on QTL locations and GP accuracies for grain sugar and mineral accumulation helps guide future breeding efforts to develop nutrient-dense and sweet cowpea varieties using indirect selection with molecular marker haplotypes. Grain sugars are mostly affected by major QTLs, therefore marker-assisted backcrossing could be employed to introduce favorable alleles from MAGIC donors to existing elite cowpea varieties. In contrast, grain mineral accumulation involved a few major but many minor QTLs, so effective breeding strategies should make use of whole-genome prediction in combination with QTL haplotype selection. For example, marker-assisted recurrent selection enables intercrosses of MAGIC lines to stack favorable haplotypes at major QTLs, followed by GP to select for lines with high genomic estimated breeding values. For multi-trait selection, priorities should be given to traits with higher GP accuracies (Fig. 7) and/or positively correlated with other traits (Fig. 2).

QTLs for sucrose and stachyose are located on separate chromosomes, thus it is possible to use molecular markers to select for high sucrose and low stachyose for improving digestibility and sweetness of cowpea. As previously described, while stachyose and other GOS improve gut health by acting as prebiotics, their fermentation by bifidobacteria and lactobacilli in the colon can also cause side effects such as diarrhea, bloating and flatulence. In a clinical study using synthetic GOS, Ito et al.^8^ reported a linear relationship between the GOS consumption and gut microbial growth, but at the optimal dose of 10 g GOS per day, stool weight and stool frequency after ingestion did not change significantly and no sign of diarrhea was observed. This dosage would be equivalent to approximately 189 g of cowpea grain containing 6% GOS and 12% moisture, for instance. Additional research may be needed to confirm whether cowpea grain derived from MAGIC lines carrying low- versus high-concentration haplotypes at the stachyose QTL may exert a significant change in gut functions. To avoid confounding effects of other nutritional factors, such research can utilize bulks of grain from MAGIC lines carrying contrasting QTL haplotype groups as described in the QTL validation section (Fig. 5). With this approach, a large number of cowpea dietary samples can be produced with varying stachyose concentrations but with equal amounts of other nutrients that potentially interact with the stachyose effect in the gut. Future investigation of candidate genes underlying the major QTLs and pathway controlling high sucrose and low stachyose content could lead to new molecular breeding approaches using gene-editing.

## Methods

### Genetic materials

Grain samples of the cowpea MAGIC population were obtained from two field sites in the USA and four in West Africa (Nigeria, Ghana, Burkina Faso and Senegal). In the USA, the population was planted at the University of California Riverside Coachella Valley Agricultural Research Station (CVARS) in Thermal, California (33.52° N, 116.15° W) under irrigation during the 2016 and 2017 autumn seasons (September–November). In West Africa, the MAGIC population was planted under rain-fed conditions during the 2017 summer season (July to October) at (1) the International Institute of Tropical Agriculture (IITA) in Minjibir, Nigeria (12.14° N, 8.66° E), (2) the Savanna Agricultural Research Institute (SARI) in Manga, Ghana (11.02° N, 0.27° W), (3) the Institut de l’Environnement et de Recherches Agricoles (INERA) in Ouagadougou, Burkina Faso (12.28° N, 1.33° W), and (4) the Institut Senegalais de Recherches Agricoles (ISRA) in the Centre National de Recherche Agronomique (CNRA), Bambey, Senegal (14.70° N, 16.45° W).

The trial at CVARS in 2016 included 291 MAGIC RILs and eight parents as described in Huynh et al.^17^. Each line was planted in a single row (0.76 m wide and 3.7 m long) at a density of 12 seeds m^-1^ using a tractor-mounted planter. The other five trials in California and West Africa each included 302 MAGIC RILs and eight parents planted in an augmented row-column (ARC) design in which 42 lines (checks) were replicated two times. The ARC designs were efficiently produced using statistical software DiGGeR package^29^. This tool searches for an optimal experimental design for the replicated check lines and then enlarges the blocks or increases the number of rows and/or columns to accommodate the un-replicated lines. The replication of check lines allows for estimation of error variance for un-replicated lines^30,31^. For the trial in California, each plot was a single row (0.76 m wide and 4.3 m long) at a density of 12 seeds m^-1^ using a tractor-mounted planter. For each plot, calendar days to flowering were determined when approximately 50% of plants in the plot flowered. At maturity, the plots were cut and allowed to dry in the field for about two weeks before threshing. A random sub-sample of harvested seeds from each plot was measured for 100-seed weight and used in nutrient assays in accordance with a sampling protocol established for cowpea^32^. Thus, there was one biological replication per variety, except 42 checks that were replicated twice in the ARC-design trial as described above. For the trials in West Africa, each plot was a single row (0.75 m wide and 2 m long row) at a density of 10 seeds m^-1^. A random sub-sample of 50 harvested seeds from each line was sent to the University of California Riverside and bulked for validation of QTL allelic effects as described later in the QTL Mapping section.

### Nutrient assays

Grain samples from Thermal, California, USA and bulked grain samples from West Africa were assayed for soluble sugars. Sugar quantifications were performed on Agilent HPLC detection system (Agilent Technologies, Santa Clara, CA, US) using an established methodology at the University of Missouri, as previously described^33^. Briefly, approximately 5 to 6 g of seed sample was finely ground using Mini-Mill (Arthur Thomas Wiley, NJ, USA) fitted with a 20-mesh screen. Fine powder was lyophilized in a 2 mL centrifuge vial for 48 h, followed by incubation at 55℃ for 1 h and then centrifugation at 250 rpm for 30 min. The supernatant was subsequently diluted with acetonitrile:water mixture of 65:35 (v/v) prior to being injected into the HPLC system. Soluble carbohydrate compositions were then calculated based on sugar standards, which included sucrose (≥ 99% CP); D-( +) raffinose pentahydrate (≥ 99% HPLC); and D-( +) stachyose hydrate (≥ 98% HPLC) from Sigma-Aldrich (St. Louis, MO, USA). These standards were prepared in HPLC-grade water with concentrations of 50, 100, 300, 500, and 1000 µg/mL, from which calibration curves were developed as illustrated in Supplementary Figure S1. One technical replication per grain sample was run together with two interval checks (certified reference Williams 82 and KB07-15 soybean flours) that were also included in each assay to monitor and ensure the consistency and accuracy of sugar assessments as previously described^33^.

Grain micro- and macronutrients of the grain samples from Thermal, California, USA were determined by inductively coupled plasma mass spectrometry (ICP–MS) at Flinders University. Approximately 10 g of seed sample was ground with a Retsch ZM200 mill (Retsch, Haan, Germany) and oven dried at 80 °C for 4 h to remove remaining moisture. Approximately 0.3 g of each ground sample was acid-digested in a closed tube as described in Wheal et al.^34^. Elemental concentrations of samples were measured using ICP-MS (8900; Agilent, Santa Clara, CA) according to the method of Palmer et al.^35^. One technical replication per grain sample was run and two replications every 30 samples to monitor and ensure reproducibility and accuracy. Any samples with Al present at > 5 µg g-1 were considered to have unacceptable levels of purported soil contamination^36^, thus resulting in their removal. Total nitrogen was determined by combustion using an Elementar Instrument. Nutrient concentrations were measured as % or ppm, while nutrient contents were calculated as nanograms per seed.

Pearson’s correlation analysis was used to examine phenotypic relationships among traits and their consistency between the 2016 and 2017 trials in California. For the 2017 trial, phenotypic data were corrected taking account of the augmented row-column design using residual maximum likelihood (REML) implemented in ASREML-R version 4^37^. These corrected phenotypic data and those from individual plots in 2016 were used in QTL and GP analyses.

### QTL mapping

Genotypic data for the MAGIC population including 32,130 single nucleotide polymorphic (SNP) markers across 11 cowpea chromosomes were derived from Huynh et al.^16^. These markers were from the 60 K-SNP Illumina iSelect BeadArray^38^ and polymorphic in the MAGIC population with a minor allele frequency > 0.05 and a successful calling rate > 90%. QTL interval mapping was performed using the ‘mpIM’ function in R/mpMap^39^. Probabilities of founder haplotypes were computed at 1-cM steps across the genome (step = 1, mrkpos = F) and fit in a linear model for each trait. A genome-wide significance threshold of 7.56 × 10^–5^ was empirically determined using the function ‘sim.sigthr’ with 1000 simulations from a null distribution. Initially, QTL were detected as peaks on a chromosome which exceeded the significance threshold. The next step involved using the ‘fit’ function in R/mpMap which incorporated all identified QTLs simultaneously. Consequently, QTL effects were estimated in a final model following the removal of peaks that no longer met the significance threshold after accounting for all other QTLs.

Allelic effects of two major QTLs for sucrose and stachyose concentrations were validated using grain samples from the four field sites in West Africa. These samples were grouped into contrasting marker-allele classes at each QTL, then bulked using a similar method described in Huynh et al.^25^. For each field site, one bulk of grain was formed for each marker-allele class, using an equal amount (2 g) of whole grain powder from each line within the class. Each bulk was well mixed and analyzed for sugars as described above. Analysis of variance (ANOVA) was performed with computer software GenStat (Release 11, VSN International Ltd.). Factors for the ANOVA model were marker-allele class and block, with each of the four field sites in West Africa considered as a block.

### Genomic prediction

The GP accuracy for each trait was estimated using a Bayesian Ridge Regression (equivalent to GBLUP) and the model was fitted using the BGLR package^21^, using phenotypic data from the MAGIC population grown at CVARS in 2016 and 2017. This accounted for across season variations and experimental designs as described previously. Genotypic data included 15,124 SNP markers from the 60 K-SNP Illumina iSelect BeadArray^38^. These markers are polymorphic in the MAGIC population^16^, and we kept markers with a minor allele frequency > 0.05 and a successful calling rate > 90%, and spaced at least 10 kb apart on the cowpea physical map^23^. For each nutrient, the computation involved 30,000 Markov Chain Monte Carlo iterations; the first 15,000 of these were discarded as burn-in, and every other 10th sample was used to compute the posterior mean. Prediction ability of the proposed model was evaluated using two cross-validation schemes (testing/training = 1/9 and 1/4) for comparison. For example, with testing/training = 1/9, 10% of the MAGIC lines (the testing set) were left out for validation, whereas the remaining 90% were used as a training set. GP accuracy was measured as Pearson’s correlation between observed and predicted phenotypic values of the testing set. We generated 20 random partitions as described therein, and for each partition we obtained the Pearson’s correlation between observed and predicted phenotypic values and obtained the average of these correlations for each trait for each validation scheme.

### Plant ethical statement

The seed samples from Africa were prepared and shipped to the USA for nutritional assays in accordance with guidelines of the import permit for small lots of seeds issued by the United States Department of Agriculture—Animal and Plant Health Inspection Service (APHIS).

### Supplementary Information

Supplementary Figure S1.

Supplementary Table S1.

Supplementary Table S2.

Supplementary Legends.

## Data Availability

The grain-nutrient dataset generated and analyzed during the current study is available in the Dryad repository, URL: 10.6086/D1FH6J.
