# Supplementary material for: Quantitative trait loci and genomic prediction for grain sugar and mineral concentrations of cowpea [Vigna unguiculata (L.) Walp.]
Source: Sci Rep. 2024 Feb 25;14:4567. doi: 10.1038/s41598-024-55214-2 (PMC10894872; doi:10.1038/s41598-024-55214-2)
Supplement: Supplementary file 2 — Supplementary Table S1. [file 41598_2024_55214_MOESM2_ESM.pdf]

Supplementary Table S1. Variation in grain sugar and mineral concentrations, flowering time and seed size in the cowpea MAGIC population and founder parents grown at the Coachella Valley Agricultural Research Station (Thermal, California) in 2016 and 2017.

		Founder parents								MAGIC lines			
Nutrient	Year	IT89KD-288	IT84S-2049	CB27	IT82E-18	SuVita-2	IT00K-1263	IT84S-2246	IT93K-503-1	Mean	Range (min – max)	CV (%)	Pearson correlation (P <0.001) between 2016 and 2017 data
Sucrose (%)	2016	2.28	1.45	2.94	2.17	5.78	1.41	1.13	2.19	2.28	0.76 – 6.88	48.48	0.86
	2017	1.73	1.26	1.78	1.90	4.12	1.08	0.99	1.86	1.80	0.57 – 6.12	46.11	
Raffinose (%)	2016	0.62	0.63	0.67	0.63	0.74	0.63	0.59	0.67	0.64	0.49 – 0.87	9.96	0.25
	2017	0.57	0.57	0.61	0.67	0.68	0.61	0.56	0.67	0.62	0.47 – 0.82	11.56	
Stachyose (%)	2016	6.32	6.04	6.15	5.77	6.55	3.19	5.07	6.10	5.28	2.47 – 6.98	16.04	0.45
	2017	5.48	4.39	4.44	5.76	5.37	4.74	4.03	5.80	5.01	2.31 – 8.36	16.17	
Nitrogen (%)	2016	3.61	3.49	3.44	4.11	3.51	3.99	3.34	3.45	3.51	2.45 – 4.68	11.47	0.70
	2017	3.73	3.92	3.71	3.86	3.48	4.17	3.77	3.60	3.85	3.02 – 4.78	8.10	
Potassium (%)	2016	1.36	1.57	1.40	1.49	1.51	1.61	1.43	1.39	1.47	1.25 – 1.77	6.60	0.76
	2017	1.27	1.52	1.30	1.41	1.32	1.46	1.35	1.28	1.37	1.14 – 1.69	7.02	
Phosphorus (%)	2016	0.45	0.51	0.48	0.48	0.40	0.55	0.45	0.48	0.48	0.35 – 0.75	10.93	0.57
	2017	0.38	0.35	0.39	0.37	0.32	0.44	0.36	0.32	0.39	0.27 – 0.59	14.37	
Magnesium (%)	2016	0.17	0.20	0.18	0.18	0.17	0.20	0.19	0.18	0.19	0.12 – 0.24	9.56	0.76
	2017	0.15	0.20	0.18	0.17	0.18	0.20	0.18	0.16	0.18	0.12 – 0.24	10.30	
Calcium (%)	2016	0.07	0.10	0.10	0.05	0.13	0.09	0.09	0.10	0.09	0.04 – 0.16	23.59	0.73
	2017	0.08	0.08	0.09	0.06	0.10	0.09	0.07	0.08	0.08	0.04 – 0.18	24.82	
Iron (ppm)	2016	53.47	74.14	60.07	58.75	46.22	60.75	43.80	48.60	58.38	36.27 – 90.98	16.98	0.60
	2017	49.96	47.84	54.17	45.59	43.79	50.73	47.11	45.68	50.75	32.46 – 81.34	17.32	
Zinc (ppm)	2016	30.06	40.54	44.75	44.21	38.92	43.21	30.52	36.85	41.49	26.46 – 71.11	13.04	0.49
	2017	31.43	27.17	35.13	29.60	32.05	33.58	27.86	25.69	32.16	23.24 – 48.55	14.66	
Manganese (ppm)	2016	9.12	8.17	10.21	20.19	9.75	11.89	9.39	10.40	9.70	5.20 – 15.03	18.28	0.63
	2017	8.23	6.87	9.67	11.20	10.79	13.05	10.21	8.95	10.72	5.63 – 15.78	16.50	
Copper (ppm)	2016	9.42	8.84	11.30	6.13	10.01	5.64	7.01	8.47	7.77	4.92 – 12.82	16.88	0.61
	2017	7.69	6.44	9.09	5.63	7.89	5.43	5.97	6.42	6.94	4.74 – 9.92	15.59	
Flowering Time (days)	2016	50	37	34	40	44	44	40	46	41	33 – 56	12.03	0.83
	2017	49	40	39	44	44	48	44	50	45	36 – 60	9.16	
Seed Size (g/100 seeds)	2016	22.7	16.1	22.6	20	19.6	20.6	19.9	15.4	18.8	10.0 – 31.6	24.29	0.88
	2017	25.8	17.5	23.7	20.3	19.4	22.7	20.5	17.7	20.6	10.9 – 36.2	25.02	
