# Supplementary material for: Quantitative trait loci and genomic prediction for grain sugar and mineral concentrations of cowpea [Vigna unguiculata (L.) Walp.]
Source: Sci Rep. 2024 Feb 25;14:4567. doi: 10.1038/s41598-024-55214-2 (PMC10894872; doi:10.1038/s41598-024-55214-2)
Supplement: Supplementary file 3 — Supplementary Table S2. [file 41598_2024_55214_MOESM3_ESM.pdf]

Supplementary Table S2. Estimates of quantitative trait loci for grain sugar and mineral concentrations and agronomic traits measured in the eight-parent cowpea MAGIC population grown at the Coachella Valley Agricultural Research Station (Thermal, California) in 2016 and 2017. The seven right-hand columns show the founder effects (± standard errors) contributed by each founder parent relative to IT93K-503-1

Sugar concentrations (%)

Trait (year)	QTL	Chr	Position (SI)	Flanking markers	Wald	P value	PctVar	IT89KD-288	IT84S-2049	CB27	IT82E-18	Suvita-2	IT00K-1263	IT84S-2246
Sucrose (2016)	*QSuc.vu-1.1*	1	48(47, 49)	2_19390 - 2_01525	251.71	0.00E+00	47.63	-0.16 ± 0.21	0.02 ± 0.19	-0.08 ± 0.24	0.12 ± 0.23	2.24 ± 0.20	-0.12 ± 0.19	-0.11 ± 0.21
	QSuc.vu-11.1	11	69(66, 70)	2_06280 - 1_1202	54.78	1.65E-09	12.63	0.78 ± 1.10	-0.29 ± 0.54	-0.43 ± 0.54	-0.47 ± 0.54	0.35 ± 0.55	-0.38 ± 0.55	0.07 ± 0.54
Sucrose (2017)	*QSuc.vu-1.1*	1	48(47, 49)	2_19390 - 2_01525	202.29	0.00E+00	38.01	-0.02 ± 0.16	-0.17 ± 0.15	-0.16 ± 0.19	-0.07 ± 0.18	1.34 ± 0.15	-0.22 ± 0.15	-0.03 ± 0.16
	QSuc.vu-11.1	11	68(66, 70)	2_11311 - 2_32285	49.57	1.76E-08	8.57	0.59 ± 0.78	-0.33 ± 0.38	-0.33 ± 0.39	-0.20 ± 0.39	0.30 ± 0.40	-0.21 ± 0.39	-0.09 ± 0.39
Raffinose (2016)	*QRaf.vu-1.1*	1	50(47, 52)	2_31884 - 2_27374	61.04	9.37E-11	17.48	-0.01 ± 0.02	0.01 ± 0.02	-0.02 ± 0.02	0.02 ± 0.02	0.08 ± 0.02	0.00 ± 0.02	-0.01 ± 0.02
	QRaf.vu-11.1	11	67(65, 70)	2_47979 - 2_48164	34.88	1.18E-05	9.63	0.07 ± 0.08	0.02 ± 0.04	-0.01 ± 0.04	-0.01 ± 0.04	0.05 ± 0.04	0.00 ± 0.04	0.01 ± 0.04
Raffinose (2017)	*QRaf.vu-1.1*	1	48(47, 51)	2_19390 - 2_01525	41.58	6.26E-07	10.32	0.00 ± 0.01	0.00 ± 0.01	0.00 ± 0.01	0.00 ± 0.01	0.04 ± 0.01	-0.01 ± 0.01	0.00 ± 0.01
	QRaf.vu-11.1	11	69(66, 70)	2_06280 - 1_1202	34.60	1.33E-05	8.27	0.08 ± 0.06	0.01 ± 0.03	0.01 ± 0.03	0.01 ± 0.03	0.05 ± 0.03	0.02 ± 0.03	0.01 ± 0.03
Stachyose (2016)	*QSta.vu-7.1*	7	42(37, 50)	2_38448 - 2_29629	39.69	1.44E-06	11.40	0.07 ± 0.20	-0.08 ± 0.20	-0.46 ± 0.21	-0.13 ± 0.24	-0.89 ± 0.21	-0.11 ± 0.21	-0.63 ± 0.19
Stachyose (2017)	*QSta.vu-7.1*	7	43(39, 45)	2_50717 - 2_08539	47.47	4.53E-08	10.59	-0.16 ± 0.17	-0.18 ± 0.17	-0.60 ± 0.17	-0.46 ± 0.20	-0.79 ± 0.17	-0.32 ± 0.18	-0.82 ± 0.16
	QSta.vu-11.1	11	68(66, 70)	2_11311 - 2_32285	42.02	5.15E-07	9.04	1.54 ± 0.91	0.07 ± 0.45	0.20 ± 0.46	0.23 ± 0.46	0.83 ± 0.46	0.41 ± 0.46	0.21 ± 0.45

Mineral concentrations (ppm, except nitrogen measured in %)

Trait (year)	QTL	Chr	Position (SI)	Flanking markers	Wald	P value	PctVar	IT89KD-288	IT84S-2049	CB27	IT82E-18	Suvita-2	IT00K-1263	IT84S-2246
Calcium (2016)	*QCa.vu-1.1*	1	56(50, 59)	2_20430 - 2_18422	36.48	5.87E-06	8.50	73.49 ± 48.73	-20.72 ± 47.58	-5.20 ± 52.38	-128.04 ± 50.38	135.94 ± 47.89	-30.76 ± 47.27	38.10 ± 50.98
	QCa.vu-6.1	6	78(73, 80)	2_09123 - 2_09998	49.40	1.89E-08	13.84	-71.84 ± 91.74	130.73 ± 71.60	106.49 ± 73.08	65.34 ± 73.65	-12.95 ± 72.48	83.56 ± 73.70	-88.03 ± 89.52
	QCa.vu-7.1	7	36(34, 44)	2_49724 - 2_51235	22.81	1.84E-03	8.57	-95.96 ± 46.99	-80.99 ± 44.93	-161.24 ± 47.99	-169.57 ± 51.95	-70.70 ± 49.35	-171.05 ± 47.25	-152.91 ± 47.69
Calcium (2017)	*QCa.vu-1.1*	1	68(57, 69)	2_23847 - 2_27947	53.82	2.55E-09	8.90	182.75 ± 46.89	-39.31 ± 41.41	-63.98 ± 41.05	-105.67 ± 40.45	55.22 ± 40.51	-7.39 ± 41.49	29.15 ± 42.84
	QCa.vu-3.1	3	7(5, 12)	2_14343 - 2_19920	35.84	7.79E-06	7.76	146.54 ± 51.68	79.91 ± 41.48	-67.95 ± 43.91	-59.21 ± 42.55	19.75 ± 39.85	-26.78 ± 45.33	3.64 ± 42.13
	QCa.vu-6.1	6	79(73, 80)	2_14712 - 2_54463	49.31	1.98E-08	11.65	-49.87 ± 78.23	120.70 ± 61.27	112.14 ± 61.33	53.53 ± 62.22	24.57 ± 61.97	113.01 ± 61.74	-60.77 ± 78.86
	QCa.vu-7.1	7	36(34, 44)	2_49724 - 2_51235	24.93	7.79E-04	7.72	-122.37 ± 41.28	-97.43 ± 40.84	-180.76 ± 40.80	-151.69 ± 45.29	-137.73 ± 44.08	-149.08 ± 42.21	-165.49 ± 42.96
Copper (2016)	*QCu.vu-1.1*	1	18(17, 19)	2_49075 - 2_48679	101.37	0.00E+00	27.09	-0.26 ± 0.29	-0.32 ± 0.27	-0.05 ± 0.32	-1.21 ± 0.27	0.24 ± 0.27	-1.58 ± 0.25	-1.57 ± 0.25
Copper (2017)	*QCu.vu-1.1*	1	18(17, 21)	2_49075 - 2_48679	150.09	0.00E+00	35.67	0.19 ± 0.21	0.28 ± 0.19	-0.35 ± 0.22	-1.05 ± 0.19	0.17 ± 0.19	-1.26 ± 0.17	-1.04 ± 0.18
	QCu.vu-9.1	9	57(50, 61)	2_05758 - 2_26862	25.40	6.44E-04	8.69	-0.77 ± 0.23	-0.57 ± 0.21	-0.69 ± 0.21	-0.23 ± 0.20	-0.26 ± 0.20	-0.65 ± 0.27	0.19 ± 0.27
Iron (2016)	*QFe.vu-1.1*	1	64(56, 68)	2_19060 - 2_37131	36.54	5.72E-06	10.76	9.01 ± 2.56	-0.96 ± 2.32	0.30 ± 2.30	-3.74 ± 2.29	-0.40 ± 2.28	-4.31 ± 2.36	-2.81 ± 2.36
	QFe.vu-7.1	7	50(47, 67)	2_23176 - 2_06992	21.08	3.66E-03	9.67	1.44 ± 2.58	6.53 ± 2.44	6.37 ± 2.44	0.78 ± 2.80	8.03 ± 2.60	4.43 ± 2.45	3.05 ± 2.23
	QFe.vu-7.2	7	84(82, 86)	2_47837 - 2_10645	29.91	9.88E-05	12.74	5.30 ± 2.41	4.20 ± 2.53	3.78 ± 2.57	-2.22 ± 2.41	7.21 ± 2.84	8.66 ± 2.69	2.43 ± 2.76
Iron (2017)	*QFe.vu-8.1*	8	26(25, 28)	2_44653 - 2_53929	30.18	8.81E-05	7.62	5.04 ± 1.87	-0.24 ± 2.24	3.93 ± 1.99	-0.17 ± 2.08	6.85 ± 2.08	0.19 ± 1.98	5.49 ± 2.03
	QFe.vu-9.1	9	6(3, 13)	2_32574 - 2_00143	36.02	7.18E-06	9.38	-12.50 ± 3.73	-9.72 ± 3.80	-11.20 ± 3.74	-8.83 ± 3.76	-11.10 ± 3.79	-14.46 ± 7.31	-3.15 ± 9.11
Magnesium (2016)	*QMg.vu-6.1*	6	41(34, 80)	2_23282 - 2_43885	23.37	1.47E-03	9.47	-82.18 ± 57.22	113.05 ± 59.94	62.32 ± 52.00	62.35 ± 56.70	28.67 ± 51.20	98.23 ± 51.67	-7.10 ± 58.57
	QMg.vu-6.2	6	78(73, 80)	2_09123 - 2_09998	19.62	6.45E-03	11.77	71.15 ± 99.85	50.70 ± 65.89	114.32 ± 66.95	48.19 ± 67.85	-8.71 ± 64.86	26.73 ± 68.90	-145.68 ± 94.16
	QMg.vu-8.1	8	43(39, 50)	2_55047 - 2_10140	40.54	9.90E-07	10.00	-2.58 ± 43.82	54.65 ± 50.04	26.07 ± 43.69	43.89 ± 46.98	-30.37 ± 44.06	143.24 ± 42.89	-98.43 ± 57.23
Magnesium (2017)	*QMg.vu-6.1*	6	53(49, 80)	2_08480 - 2_11761	34.05	1.68E-05	17.95	-126.93 ± 85.98	145.06 ± 74.52	87.03 ± 72.23	91.78 ± 75.51	125.26 ± 76.25	29.44 ± 71.00	42.25 ± 85.72
	QMg.vu-6.2	6	79(77, 80)	2_14712 - 2_54463	33.92	1.79E-05	20.58	103.56 ± 109.18	121.56 ± 63.98	121.84 ± 65.83	88.13 ± 65.21	4.72 ± 63.61	153.90 ± 65.28	-140.81 ± 104.30
	QMg.vu-8.1	8	43(41, 51)	2_55047 - 2_10140	38.77	2.17E-06	8.51	-52.47 ± 42.16	21.92 ± 47.48	-5.02 ± 42.12	24.19 ± 44.18	-0.58 ± 42.14	128.61 ± 40.80	-75.48 ± 55.25

Manganese (2016)	*QMn.vu-5.1*	5	5(4, 6)	2_16237 - 2_39065	79.80	1.51E-14	22.28	0.29 ± 1.30	-2.81 ± 0.72	-0.81 ± 0.72	0.57 ± 0.68	-0.52 ± 0.70	-0.83 ± 0.69	-0.19 ± 0.74
Manganese (2017)	*QMn.vu-1.1*	1	27(23, 35)	2_25838 - 2_21671	38.37	2.57E-06	10.42	-0.07 ± 0.32	-0.19 ± 0.29	-1.28 ± 0.37	0.56 ± 0.30	-0.90 ± 0.29	-0.14 ± 0.28	0.38 ± 0.28
	QMn.vu-3.1	3	26(23, 33)	2_06927 - 2_32071	29.56	1.14E-04	9.53	0.63 ± 0.45	-0.39 ± 0.36	-0.62 ± 0.37	-0.46 ± 0.35	0.63 ± 0.36	-0.36 ± 0.35	-0.11 ± 0.44
	QMn.vu-5.1	5	5(4, 6)	2_16237 - 2_39065	124.72	0.00E+00	28.22	-0.44 ± 0.94	-3.27 ± 0.53	0.24 ± 0.52	-0.16 ± 0.50	-0.59 ± 0.51	-0.67 ± 0.51	-0.29 ± 0.55
	QMn.vu-6.1	6	80(76, 80)	2_27095 - 2_27096	63.12	3.58E-11	14.18	0.66 ± 0.61	1.71 ± 0.47	1.56 ± 0.47	1.63 ± 0.48	1.05 ± 0.48	1.14 ± 0.48	-0.01 ± 0.61
Nitrogen (2016)	*QN.vu-7.1*	7	102(96, 104)	2_23308 - 2_12667	28.91	1.50E-04	8.47	0.23 ± 0.11	0.31 ± 0.09	-0.04 ± 0.13	-0.03 ± 0.09	0.28 ± 0.14	0.38 ± 0.13	0.11 ± 0.08
	QN.vu-8.1	8	74(72, 78)	2_47201 - 2_51598	33.41	2.22E-05	9.98	-0.02 ± 0.22	-0.01 ± 0.22	-0.19 ± 0.22	0.16 ± 0.22	-0.03 ± 0.22	0.25 ± 0.22	-0.11 ± 0.41
Nitrogen (2017)	*QN.vu-3.1*	3	20(18, 38)	2_18503 - 2_07341	23.80	1.24E-03	7.90	0.14 ± 0.09	-0.11 ± 0.07	-0.02 ± 0.08	-0.12 ± 0.07	0.05 ± 0.07	-0.12 ± 0.07	-0.02 ± 0.09
	QN.vu-6.1	6	79(76, 80)	2_14712 - 2_54463	32.16	3.80E-05	9.76	0.12 ± 0.13	0.16 ± 0.10	0.25 ± 0.10	0.19 ± 0.10	0.10 ± 0.10	0.29 ± 0.10	-0.02 ± 0.13
	QN.vu-11.1	11	63(61, 67)	2_09388 - 1_0867	32.64	3.09E-05	9.84	-0.52 ± 0.27	-0.08 ± 0.15	-0.32 ± 0.15	-0.15 ± 0.15	-0.28 ± 0.15	-0.07 ± 0.15	-0.28 ± 0.15
Phosphorus (2017)	*QP.vu-2.1*	2	30(27, 32)	2_30438 - 2_41401	32.97	2.68E-05	7.88	-79.72 ± 320.65	244.11 ± 124.12	64.31 ± 140.64	111.94 ± 120.81	422.23 ± 125.82	-149.68 ± 128.00	-26.52 ± 306.26
	QP.vu-8.1	8	74(66, 78)	2_47201 - 2_51598	43.30	2.92E-07	10.90	-159.31 ± 291.67	-8.46 ± 295.82	-161.06 ± 285.36	237.71 ± 289.97	106.84 ± 291.80	359.23 ± 287.80	-316.25 ± 542.64
Potassium (2016)	*QK.vu-6.1*	6	77(73, 80)	2_30711 - 2_18464	35.96	7.37E-06	10.24	-147.26 ± 446.43	721.17 ± 357.86	727.74 ± 358.74	743.25 ± 366.97	297.92 ± 361.23	913.84 ± 367.06	215.30 ± 443.29
Potassium (2017)	*QK.vu-1.1*	1	14(10, 19)	2_46580 - 2_45982	25.96	5.13E-04	7.85	689.81 ± 227.61	229.40 ± 206.04	49.74 ± 226.28	621.38 ± 203.43	-69.99 ± 200.92	552.86 ± 181.65	170.98 ± 180.02
	QK.vu-6.1	6	80(76, 80)	2_27095 - 2_27096	39.79	1.38E-06	12.78	-118.01 ± 421.12	808.98 ± 325.75	744.65 ± 327.38	828.56 ± 328.84	623.33 ± 326.84	1099.38 ± 332.14	626.28 ± 422.66
Zinc (2017)	*QZn.vu-2.1*	2	30(23, 42)	2_30438 - 2_41401	18.72	9.11E-03	7.63	-0.04 ± 2.67	1.75 ± 1.06	0.00 ± 1.20	0.57 ± 1.02	2.61 ± 1.08	-0.92 ± 1.08	-0.67 ± 2.54
	QZn.vu-3.1	3	33(27, 36)	2_47830 - 2_52602	35.65	8.46E-06	8.98	1.92 ± 1.75	0.78 ± 1.22	1.27 ± 1.23	-1.15 ± 1.25	4.20 ± 1.25	-0.88 ± 1.20	-0.20 ± 1.41
	QZn.vu-7.1	7	67(65, 74)	2_15742 - 2_18100	33.37	2.26E-05	8.88	1.95 ± 1.24	1.60 ± 1.06	4.23 ± 1.17	0.33 ± 1.17	4.58 ± 1.27	4.21 ± 1.20	1.77 ± 1.05

Agronomic Traits (days to flowering and 100-seed weight in grams)

Trait (year)	QTL	Chr	Position (SI)	Flanking markers	Wald	P value	PctVar	IT89KD-288	IT84S-2049	CB27	IT82E-18	Suvita-2	IT00K-1263	IT84S-2246
Flowering (2016)	*QFlo.vu-1.1*	1	64(54, 69)	2_19060 - 2_37131	37.23	4.25E-06	13.50	0.55 ± 1.05	-2.09 ± 0.95	-2.55 ± 0.96	-2.22 ± 0.94	-1.15 ± 0.90	1.56 ± 0.95	-1.98 ± 0.96
	QFlo.vu-4.1	4	18(14, 27)	2_44685 - 2_27063	65.60	1.14E-11	12.59	-1.58 ± 2.93	0.79 ± 1.80	-3.79 ± 1.73	1.15 ± 1.83	0.92 ± 1.78	2.27 ± 1.75	0.26 ± 2.23
	QFlo.vu-5.1	5	8(5, 16)	2_32176 - 2_18349	40.73	9.13E-07	10.34	4.37 ± 2.49	-0.08 ± 1.45	-1.83 ± 1.42	0.75 ± 1.36	-0.78 ± 1.37	-1.07 ± 1.37	1.95 ± 1.43
	QFlo.vu-9.1	9	10(8, 14)	2_14794 - 2_20854	68.24	3.35E-12	17.43	-0.34 ± 1.57	0.44 ± 1.58	-3.72 ± 1.59	-1.63 ± 1.61	-0.16 ± 1.57	2.71 ± 3.43	2.59 ± 3.97
	QFlo.vu-11.1	11	56(45, 58)	2_03157 - 2_54173	33.87	1.82E-05	10.00	6.76 ± 3.62	6.16 ± 1.99	1.16 ± 1.97	4.15 ± 1.96	2.29 ± 1.98	3.39 ± 1.97	2.81 ± 1.96
Flowering (2017)	*QFlo.vu-4.1*	4	20(16, 37)	2_31776 - 2_15171	39.71	1.43E-06	9.32	-5.26 ± 2.57	-0.95 ± 1.63	-5.38 ± 1.54	-1.62 ± 1.59	-3.07 ± 1.58	-1.41 ± 1.55	-3.49 ± 2.00
	QFlo.vu-9.1	9	11(4, 14)	2_38670 - 2_47664	50.33	1.25E-08	12.31	-0.67 ± 1.52	0.47 ± 1.54	-2.57 ± 1.53	-0.30 ± 1.55	-0.61 ± 1.53	2.19 ± 3.26	3.48 ± 3.64
Seed size (2016)	*QSdw.vu-6.1*	6	79(73, 80)	2_14712 - 2_54463	31.84	4.35E-05	8.14	0.48 ± 1.69	-2.31 ± 1.36	-2.81 ± 1.35	-2.82 ± 1.35	-0.68 ± 1.34	-2.09 ± 1.36	-0.56 ± 1.73
	QSdw.vu-8.1	8	74(73, 76)	2_47201 - 2_51598	158.94	0.00E+00	36.14	2.22 ± 1.99	2.52 ± 2.02	3.67 ± 1.95	9.19 ± 1.99	2.20 ± 2.01	8.23 ± 1.98	4.15 ± 3.70
Seed size (2017)	*QSdw.vu-6.1*	6	80(75, 80)	2_27095 - 2_27096	44.60	1.63E-07	9.28	0.35 ± 1.86	-3.50 ± 1.49	-3.67 ± 1.46	-3.43 ± 1.49	-1.27 ± 1.47	-3.53 ± 1.49	-0.78 ± 1.92
	QSdw.vu-8.1	8	74(73, 77)	2_47201 - 2_51598	175.82	0.00E+00	35.86	4.22 ± 2.23	4.27 ± 2.26	5.21 ± 2.18	10.21 ± 2.22	3.58 ± 2.24	11.13 ± 2.20	5.76 ± 4.15

Chr, chromosome; Position, position in centimorgans (and 1-LOD support interval, SI); PctVar, percentage of variance explained; MAGIC, multi-parent advanced generation inter-cross.
