# Supplementary material for: Quantitative trait loci and genomic prediction for grain sugar and mineral concentrations of cowpea [Vigna unguiculata (L.) Walp.]
Source: Sci Rep. 2024 Feb 25;14:4567. doi: 10.1038/s41598-024-55214-2 (PMC10894872; doi:10.1038/s41598-024-55214-2)
Supplement: Supplementary file 4 — Supplementary Legends. [file 41598_2024_55214_MOESM4_ESM.docx]

**Supplementary Table and Figure Legends**

**Table S1.** Variation in grain sugar and mineral concentrations, flowering time and seed size in the cowpea MAGIC population grown at CVARS in 2016 and 2017.

**Table S2.** Estimates of quantitative trait loci for grain sugar and mineral concentrations and agronomic traits measured in the cowpea MAGIC population grown at CVARS in 2016 and 2017.

**Figure S1**. Calibration curves of sugar standards (sucrose, raffinose and stachyose) measured at different concentrations (50, 100, 300, 500, and 1000 µg/mL) by high-performance liquid chromatography. Values were converted to natural logarithm (Ln).
